# DNA Double Strand Breaks as Predictor of Efficacy of the Alpha-Particle Emitter Ac-225 and the Electron Emitter Lu-177 for Somatostatin Receptor Targeted Radiotherapy

**DOI:** 10.1371/journal.pone.0088239

**Published:** 2014-02-07

**Authors:** Franziska Graf, Jörg Fahrer, Stephan Maus, Alfred Morgenstern, Frank Bruchertseifer, Senthil Venkatachalam, Christian Fottner, Matthias M. Weber, Johannes Huelsenbeck, Mathias Schreckenberger, Bernd Kaina, Matthias Miederer

**Affiliations:** 1 University Medical Centre, Department of Nuclear Medicine, Mainz, Germany; 2 University Medical Centre, Institute of Toxicology, Mainz, Germany; 3 European Commission**,** Joint Research Centre – Institute for Transuranium Elements, Karlsruhe, Germany; 4 University Medical Centre, Department of Endocrinology, Mainz, Germany; University of Pittsburgh, United States of America

## Abstract

**Rationale:**

Key biologic effects of the alpha-particle emitter Actinium-225 in comparison to the beta-particle emitter Lutetium-177 labeled somatostatin-analogue DOTATOC *in vitro* and *in vivo* were studied to evaluate the significance of γH2AX-foci formation.

**Methods:**

To determine the relative biological effectiveness (RBE) between the two isotopes (as - biological consequence of different ionisation-densities along a particle-track), somatostatin expressing AR42J cells were incubated with Ac-225-DOTATOC and Lu-177-DOTATOC up to 48 h and viability was analyzed using the MTT assay. DNA double strand breaks (DSB) were quantified by immunofluorescence staining of γH2AX-foci. Cell cycle was analyzed by flow cytometry. *In vivo* uptake of both radiolabeled somatostatin-analogues into subcutaneously growing AR42J tumors and the number of cells displaying γH2AX-foci were measured. Therapeutic efficacy was assayed by monitoring tumor growth after treatment with activities estimated from *in vitro* cytotoxicity.

**Results:**

Ac-225-DOTATOC resulted in ED_50_ values of 14 kBq/ml after 48 h, whereas Lu-177-DOTATOC displayed ED_50_ values of 10 MBq/ml. The number of DSB grew with increasing concentration of Ac-225-DOTATOC and similarly with Lu-177-DOTATOC when applying a factor of 700-fold higher activity compared to Ac-225. Already 24 h after incubation with 2.5–10 kBq/ml, Ac-225-DOTATOC cell-cycle studies showed up to a 60% increase in the percentage of tumor cells in G2/M phase. After 72 h an apoptotic subG1 peak was also detectable. Tumor uptake for both radio peptides at 48 h was identical (7.5%ID/g), though the overall number of cells with γH2AX-foci was higher in tumors treated with 48 kBq Ac-225-DOTATOC compared to tumors treated with 30 MBq Lu-177-DOTATOC (35% vs. 21%). Tumors with a volume of 0.34 ml reached delayed exponential tumor growth after 25 days (44 kBq Ac-225-DOTATOC) and after 21 days (34 MBq Lu-177-DOTATOC).

**Conclusion:**

γH2AX-foci formation, triggered by beta- and alpha-irradiation, is an early key parameter in predicting response to internal radiotherapy.

## Introduction

The clinical impact of tumor-targeted radionuclide therapy, primarily using beta-particle emitters, is growing and treatment methods for metastasized malignancies with unfavorable prognosis have been developed. In patients with metastasized neuroendocrine tumors, high somatostatin receptor status provides the opportunity for peptide receptor radionuclide therapy (PRRT) with commonly used somatostatin analogues, e. g., octreotide, DOTATOC and DOTATATE, radiolabelled with the beta-emitting nuclide Lutetium-177 (^177^Lu, half-life 6.73 d, 0.498 MeV) leading to tumor regression and symptom reduction [1]. Nevertheless, kidney and hematologic toxicities after PRRT have been reported [2]. Progressive disease and early relapses in patients with radio-resistant tumors were also described.

To further improve the PRRT strategy for neuroendocrine tumors, labeling of somatostatin analogues with an alpha-particle emitter could be an attractive option. Alpha-particle emitters are characterized by a high energy and high linear energy transfer (LET) causing high cellular cytotoxicity at the site of radionuclide decay [3]. Compared to beta-particles and gamma irradiation the higher LET of alpha-particles leads to denser ionisation events along the particle track. This, in turn leads to a higher fraction of double strand breaks per track length and therefore a higher biological effectiveness. Thus, the same energy transferred to tissue is more toxic for alpha particles than for beta particles and a respective factor, the relative biological effectiveness (RBE) has to be taken into account to enable comparability between doses from different radiation types. This RBE is known to be in the range of 5–10 for alpha-particles over beta-particles. Furthermore, the short range of alpha-particles (<100 microns) is promising for treatment of tumor micrometastases and reduction of side effects in healthy tissue. Indeed, efficacy of tumor-specific antibodies and peptides labelled with alpha-particle emitting nuclides, e. g., Actinium-225 (^225^Ac) and Bismuth-213 (^213^Bi) were described *in vitro* and *in vivo* [4], [5].

^225^Ac (half-life 9.9 d, 5.935 MeV) has six daughters in its decay chain – generating in total four alpha-particles – finally resulting in stable Bismuth-209 at the end [6]. After ^221^Fr and ^217^At, ^213^Bi (half-life 46 min) is one daughter of ^225^Ac that decays mainly (98%) by beta-emission, producing an alpha-emitter ^213^Po (half-life 4.2 µs, 8.375 MeV), but also has a direct alpha-emission (2%, 5.87 MeV) to ^209^Tl (half-life 2.2 min, 3.98 MeV). Because of the longer half-life and numerous alpha-particle emissions, radiotherapy with ^225^Ac has been reported to be much more cytotoxic compared to the very short lived alpha emitter ^213^Bi (half live 46 min) [7]. For example, the tumor-homing peptide, F3, labelled with the alpha emitters ^213^Bi- and ^225^Ac significantly prolonged survival of mice with peritoneal carcinomatosis along with the absence of severe toxic side effects [8]. Also, therapeutic efficacy and nephrotoxicity of ^225^Ac-DOTATOC has been studied in a preclinical mouse model of neuroendocrine tumors where a therapeutic window between tumor efficacy and long-term toxicity was demonstrated [9].

Despite promising results of alpha-targeted therapy, few data are available about the biological and molecular mechanisms of tumor cell damage. In particular, a number of distinct cellular effects occur between an ionization event and final cell death. Within these pathways, for example, cellular properties like radiation sensitivity or resistance are determined. Apart from radiation properties like LET and range in tissue, dose rates may also have a major impact on biologic responses. Therefore, dose-rate effects should be considered when comparing radioisotopes that vary significantly in terms of their physical half-life. Furthermore, variations in the physical properties of radiation may provoke different cellular responses depending on the relevant pathways, e.g., cell death and DNA repair mechanisms. In multiple resistant leukemia and non-Hodgkin Lymphoma cells, re-activation of apoptotic pathways were reported to be key mechanisms of alpha radiation for overcoming treatment resistance [10], [11].

The comparison of alpha- and beta-particle emitters with similar half-lives at equitoxic doses could provide new insights into the cellular response after high and low-LET radiation. The aim of the study is to compare the alpha-particle emitter ^225^Ac- and beta emitter ^177^Lu-labelled somatostatin analogue DOTATOC in terms of DNA damage induction and the level of cell death. We studied this in the neuroendocrine cell line AR42J, which is characterized by expression of wild-type p53 and strong somatostatin expression of target epitopes, where intact apoptotic pathways have been demonstrated [12]. We determined the level of γH2AX as a reliable and sensitive indicator of DNA double-strand breaks (DSB) and apoptosis, which is the main mechanism of cell death following treatment with the radioisotopes.

## Materials and Methods

### Material

The antibodies against Hsp90, p21 and p53 were bought from Santa Cruz Biotechnology (Heidelberg, Germany). The antibody against ATM was obtained from Cell Signaling (Boston, MA, USA) and the antibody against phosphorylated ATM was from Merck Millipore (Darmstadt, Germany). PARP-1 antibody was a kind gift of Prof. Dr. Alexander Bürkle (University of Konstanz, Germany).

### Chemistry

#### Radiolabeling of somatostatin analogues

All chemicals and the peptide DOTATOC were obtained from commercial sources.

^225^Ac was produced at the European Commission Joint Research Centre, Institute for Transuranium Elements, Karlsruhe, Germany [13]. ^225^Ac was quantified with a gamma counter using the gamma emissions of its daughter nuclides ^221^Fr (half-life: 4.9 min) and ^213^Bi (half-life: 46.6 min) using a 190–247 keV and 399–488 keV energy window, respectively, after radiochemical equilibrium was reached. The synthesis starting from 10 µl of DOTATOC (jpt peptides, Germany) (0.5 mg/ml) and 1–2 MBq ^225^Ac (0.1 M HCl) in 0.1 M Tris buffer pH 9.0 yielded ^225^Ac-DOTATOC within 30 min at 90°C and a radiochemical purity of typically >95% assessed via ITLC. The specific activity of ^225^Ac-DOTATOC was calculated to 0.2–0.4 MBq/µg.

^177^Lu was kindly provided by IDB Holland, Baarle-Nassau, The Netherlands, and counted in a calibrated dose activimeter or in a gamma counter using an energy window of 126–159 keV. For radiolabeling of DOTATOC, 200 µl of ^177^LuCl_3_ solution (4 GBq; IDB Holland, Baarle-Nassau, The Netherlands) was incubated with 100 µg DOTATOC in 400 µl 0.4 M sodium acetate buffer with gentisic acid (2,5-dihydroxy benzoic acid, 25 mg/ml) at 95°C for 30 min. Radiochemical purity was >99% and specific activity ∼40 MBq/µg.

### Cellular Studies

#### Cell culture

The p53 wild-type tumor cell line AR42J, a rat pancreatic acinar carcinoma cell line, was obtained from ATCC-LGC Standards, Wesel, Germany and cultured in DMEM (high glucose, 4.5 g/l; PAA, Pasching, Austria) supplemented with 10% fetal bovine serum (FBS) and 1% penicillin-streptomycin. The cells were cultured at 37°C, 5% CO_2_ and 95% humidity in a CO_2_ incubator (Heracell, Heraeus, Hanau, Germany; Binder, Germany).

#### SDS-PAGE and western blot analysis

Following irradiation with 5 Gy, cells were harvested in 1x Lämmli loading buffer. Samples were then subjected to SDS-PAGE followed by transfer onto a nitrocellulose membrane (Whatman, Dassel, Germany) using a wet-blot chamber (GE Healthcare, München, Germany). The membrane was blocked with 5% (w/v) non-fat dry milk in PBS containing Tween-20 [0.1% (v/v), PBS-T] for 1 h at room temperature (RT). Subsequently, the membrane was incubated with the respective primary antibody diluted in PBS-T for 1 h at RT. After washing the membrane, it was incubated with the appropriate secondary antibody coupled to horseradish peroxidase (Santa Cruz Biotechnology, Heidelberg, Germany) for 1 h. After further washing steps, bound antibodies were visualized by chemiluminescence detection using Western Lightning® Plus-ECL (Perkin Elmer, Rodgau, Germany).

#### Cell viability

Cell viability was analyzed for up to 48 h after treatment of ^225^Ac-DOTATOC or ^177^Lu-DOTATOC, respectively. 2×10^4^ cells were seeded into a 96 well cell culture plate and incubated for 24 h. Next, respective compound was added in increasing concentrations (0.001 to 250 kBq/ml ^225^Ac-DOTATOC and 5 to 40,000 kBq/ml ^177^Lu-DOTATOC) to the cells for 24 h and 48 h. Incubation of tetrazolium salt MTT was performed at a final concentration of 50 µg/ml at 37°C in the dark for 60 min. After that, supernatant was completely removed, cells were lyzed with 100 µl DMSO and absorption was detected at 550 nm and 690 nm as a reference wavelength.

#### Alkaline comet assay

Formation of DNA damage after exposure to ^225^Ac-DOTATOC and ^177^Lu-DOTATOC, respectively, was assayed by single-cell gel electrophoresis after alkaline cell lysis as described previously [14],[15]. Agarose embedded cells on a slide were first incubated in lysis buffer (2.5 M NaCl, 100 mM EDTA, 10 mM Tris, 1% sodium lauroylsarcosinate, pH 10) for 1 h at 4°C. After a second incubation period in 300 mM NaOH and 1 mM EDTA (pH>13) for 15 minutes at 4°C, electrophoresis (25 V/300 mA) was carried out for 15 minutes at 4°C. Cells were fixed in 100% ethanol and stained with propidium iodide (PI). The DNA comets were visualized with a fluorescence microscope and quantified by determination of the tail moment (percentage of DNA in the tail multiplied by the length between the center of the head and tail) [16] using Komet 4.0.2 software (Kinetic Imaging Ltd., Merseyside, UK). Per treatment 50 nuclei in total were measured (mean ± standard deviation from at least three independent experiments).

#### γH2AX phosphorylation *in vitro*

Cells were seeded onto cover slides. After treatment with ^225^Ac-DOTATOC or ^177^Lu-DOTATOC, cells were fixed with 4% paraformaldehyde for 15 min at room temperature, following incubation with ice-cold methanol (−20°C, 10 min). After blocking with PBS containing 0.3% Triton-X-100 and 5% BSA (w/v) (1 h, room temperature) incubation with Anti-phospho-Histone H2A.X(Ser139) mouse monoclonal antibody (1∶750, Millipore) was conducted overnight at 4°C. Incubation with the secondary fluorophore-labelled antibody (AlexaFluor 488 goat anti-mouse, 1∶1000, Invitrogen) was performed for 1 h at room temperature in the dark. For immunofluorescence microscopy of γH2AX a Zeiss Imager.M1 (Carl Zeiss AG, Oberkochen, Germany) was used. Values are given as mean ± standard deviation from at least three independent experiments with 50 nuclei each being analyzed.

#### Cell cycle measurements

Cell cycle distribution of the cells was determined by flow cytometry DNA analysis. After treatment of 2.5×10^5^ cells for 24, 48, 72 and 96 h with ^225^Ac-DOTATOC or ^177^Lu-DOTATOC, respectively, cells were washed with PBS and detached from the cell culture flask by addition of trypsin. After centrifugation for 5 min at 1500 rpm at 4°C, cells were washed once with ice-cold PBS and fixed in 70% ethanol at 20°C for at least 60 min. Ethanol-fixed cells were washed again, treated with 1 µg/ml ribonuclease I (Sigma-Aldrich) for 60 min and stained with 10 µg/ml propidium iodide (Sigma-Aldrich) in the dark. Flow cytometry analysis was performed on a FACSCalibur™ Flow Cytometer (Becton Dickinson, Heidelberg, Germany) by use of the CellQuest Pro software. For cell cycle analysis, 10,000 events were collected in the single-events region with a total event rate not exceeding 300 events/second.

#### Apoptosis quantification

Trypsinized adherent cells were resuspended in cold PBS and then fixed in ice-cold 70% ethanol for a minimum of 60 min. After RNase (1 µg/ml) digestion for one hour DNA was stained with propidium iodide (PI; 10 µg/ml) in PBS. For each sample 10,000 cells were subjected to flow cytometric analysis using a FACSCalibur™ Flow Cytometer (Becton Dickinson, Heidelberg, Germany). The number of apoptotic cells (subG_1_ fraction) was calculated using the software WinMDI 2.9.

### *In vivo* Experiments

#### Treatment of AR42J-xenograft tumors in nude mice

All animal procedures and experiments were carried out according to the guidelines of the German Regulations for Animal Welfare. The protocols were approved by the local Ethical Committee for Animal Experiments (Landesuntersuchungsamt Rheinland-Pfalz, 23 177-07/G10-1-013).

BALB/c nu/nu mice (Charles River) with an age of 9–10 weeks and an average weight of 20 g were injected subcutaneously with 5·10^6^ AR42J cells into the right flank and randomly divided into groups of 2–3 animals. After the xenograft tumor reached 0.5 cm (14 days post injection) in diameter either 47 kBq of ^225^Ac-DOTATOC or 30 MBq of ^177^Lu-DOTATOC were intravenously injected into the tail vain. The reference groups analogously received 0.9% sodium chloride solution, or respectively, 1 µg of unlabelled DOTATOC (n = 3). 48 h after treatment, all mice were sacrificed and tumor and major organs (kidney, liver, lung, heart, muscle) were dissected. The weight and activity were measured for each tissue section. Tumor samples were fixed in 4% formalin and paraffin embedded for histological analyses.

In a second experiment, mice carrying AR42J tumors of approximately 0.3 cm^3^ were treated intravenously with either 44 kBq (±3.5, n = 3) ^225^Ac-DOTATOC or with 34 MBq (±4.1, n = 4) ^177^Lu-DOTATOC with a single injection and compared to growth control (n = 4). After treatment tumors were measured in two dimensions trice weekly with calipers until exponential growth phase was clearly reached and mice were then sacrificed. For each time point, the mean tumor sizes from all animals in one group were calculated and plotted over time. Statistical analyses were performed using SPSS, version 20 (SPSS Inc., Chicago, IL, USA) with a linear model for repeated measurements to compare development of tumor size within the groups over time. Until seven days after treatment comparison of three groups and until 23 days comparison of both verum groups was analysed.

#### γH2AX phosphorylation in tissue sections

Formalin fixed paraffin embedded AR42J tumors were cut at 5 µm sections and immunohistochemically analyzed for γH2AX levels.

A standard immunohistochemical technique was performed using the rabbit monoclonal antibody to phospho-histone γH2AX (1∶400). Therefore, sections were deparaffinized in xylene and rehydrated via graded ethanol and PBS. Heat epitope retrieval was done at 95°C for 60 min in DAKO Target retrieval solution (DAKO, Hamburg, Germany), followed by annealing at room temperature for 20 min. Nonspecific binding sites were blocked with DAKO Protein Blocking Solution (DAKO, Hamburg, Germany). The primary antibody (1∶400; Abcam, Ambridge, UK) was incubated overnight at 4°C in PBS with 0.2% Triton X-100. Incubation of the Alexa488-coupled secondary antibody (1∶600; Life Technologies, Darmstadt, Germany) was performed for 2 h followed by DNA staining with TOPRO-3 dye (1∶100; Life Technologies, Darmstadt, Germany for 30 min at room temperature in the dark. Slides were mounted with Vectashield medium (Linaris, Dossenheim, Germany) and analyzed by confocal microscopy with a Zeiss Axio Observer.Z1 microscope equipped with a LSM710 laser-scanning unit (Zeiss, Oberkochen, Germany). Images were acquired in optical sections of 1 µm and processed with ImageJ (NIH, USA). Necrotic tissue areas were not included in the interpretation of immunostaining. The percentage of γH2AX -positive cells was scored in at least 8 different sections of each tumor (∼40 cells per section) and data was evaluated by GraphPad Prism software.

## Results

### DNA Damage Response in AR42J Cells

**Figure 1:**
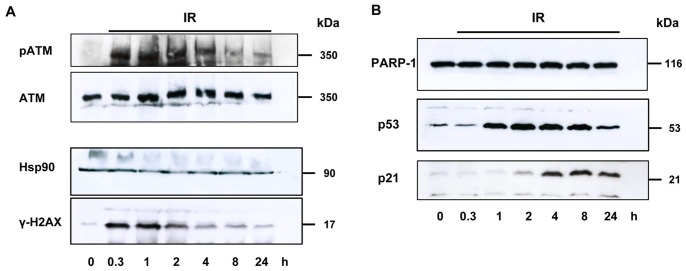
DNA damage response in AR42J cells following ionizing irradiation. (A) and (B) Cells were irradiated with 5 Gy and time-dependent DNA damage response was assessed over 24 h using western blot analysis.

First, we characterized the DNA damage response (DDR) of AR42J pancreatic acinar carcinoma cells following ionizing radiation. Cells were subjected to gamma irradiation (5 Gy), which is a well-described inducer of DNA double strand breaks (DSBs) [17], and incubated for up to 24 h. Western blot analysis showed a fast and strong phosphorylation of ATM, a phosphatidylinositol 3-kinase-related kinase (PIKK) that governs the cellular stress response to DSBs (Fig. 1A, top panel). This is consistent with the notion that ATM is activated upon irradiation by autophosphorylation [18], resulting in the stimulation of ATM-mediated DSB repair [19]. The IR-dependent activation of ATM in AR42J cells was accompanied by phosphorylation of its substrate histone 2AX (γH2AX), which is an established marker for DSBs [20] (Fig. 1A, bottom panel). As expected, γH2AX peaked 0.3–1 h upon IR and returned to baseline levels after 24 h, reflecting the formation and repair of DSBs. In addition, we analyzed the irradiation-mediated response of p53 and observed its time-dependent accumulation, starting 1 h after IR (Fig. 1B). This was followed by induction of its downstream target p21 that is capable of inducing cell cycle arrest. In contrast, the levels of the DNA repair protein PARP-1 remained unchanged upon IR.

Taken together, these findings show that AR42J cells activate the DDR in an ATM-dependent manner following gamma irradiation and are proficient in p53 signaling as attested by its accumulation and subsequent induction of p21.

### Cell Viability Studies

**Figure 2:**
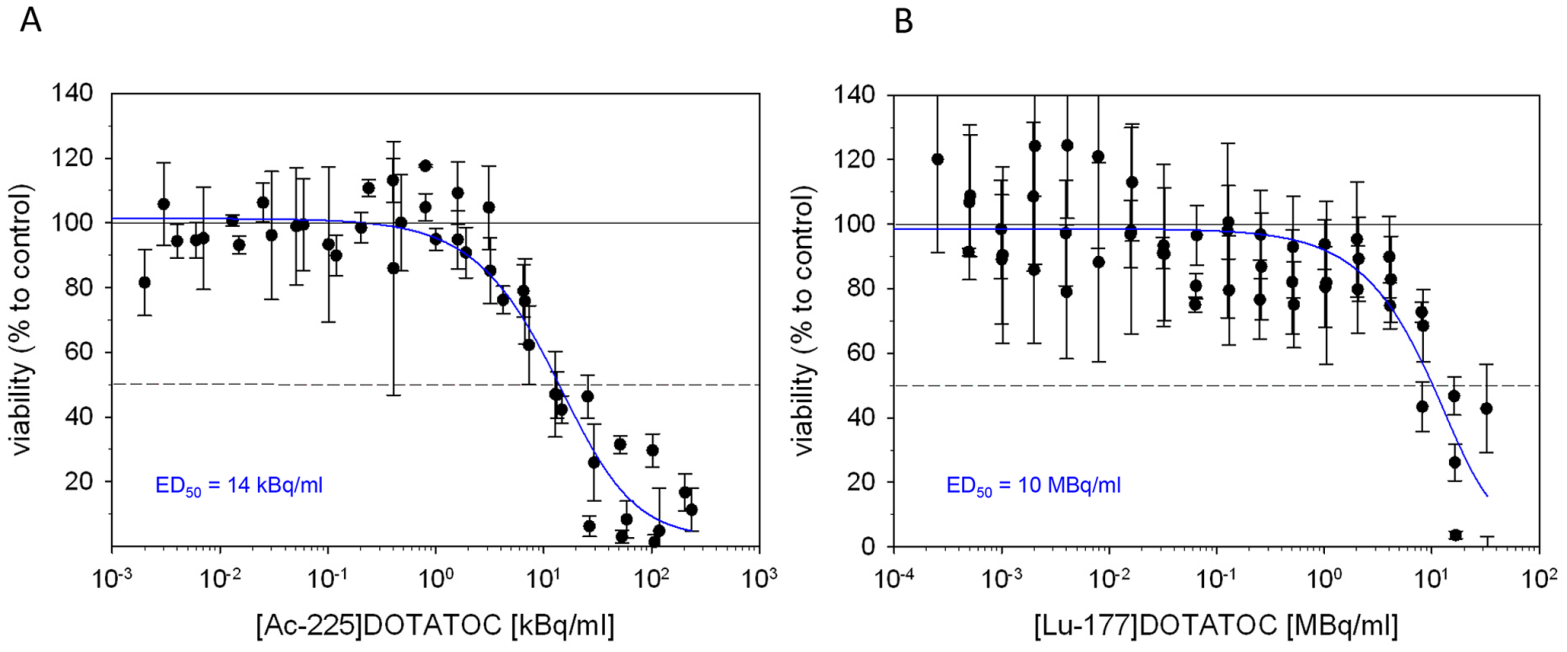
Viability of AR42J cells at 48^225^Ac-DOTATOC (A) and ^177^Lu-DOTATOC (B).

Viability of pancreatic tumor cells after treatment with ^225^Ac-DOTATOC and ^177^Lu-DOTATOC was measured with the MTT assay. The colorimetric assay is dependent on the enzymatic activity of intracellular oxidoreductase that metabolizes the terazolium dye MTT and reflects the number of viable cells. Cellular studies showed a reduced viability of AR42J cells after incubation with ^225^Ac-DOTATOC at activity concentrations greater than 2–4 kBq/ml. ED_50_ values were calculated to 30 kBq/ml after 24 h and 14 kBq/ml after 48 h (Fig. 2). The same effect was found for ^177^Lu-DOTATOC, but only at about 700-fold higher activities. 48 h after incubation with ^177^Lu-DOTATOC ED_50_ value was calculated to 10 MBq/ml (Fig. 2).

### DNA Damage

**Figure 3:**
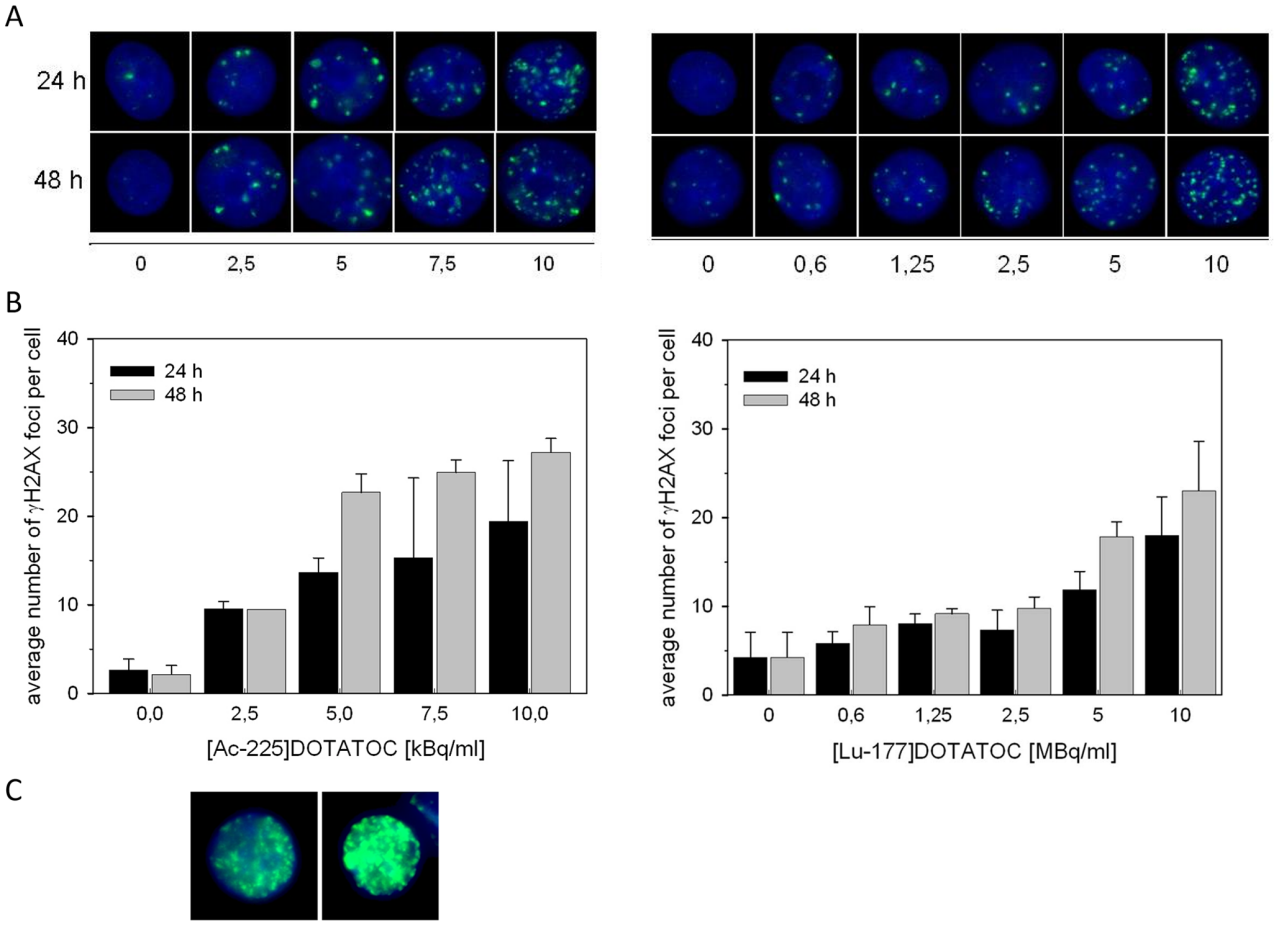
Number of γH2AX foci in AR42J cells at 24 and 48 h after incubation with ^225^Ac-DOTATOC (left) and ^177^Lu-DOTATOC (right). (A) shows representative images from all activity levels, (B) shows quantification of γH2AX foci and (C) shows two representative examples for pan nuclear staining after high dose ^225^Ac-DOTATOC treatment.

**Figure 4:**
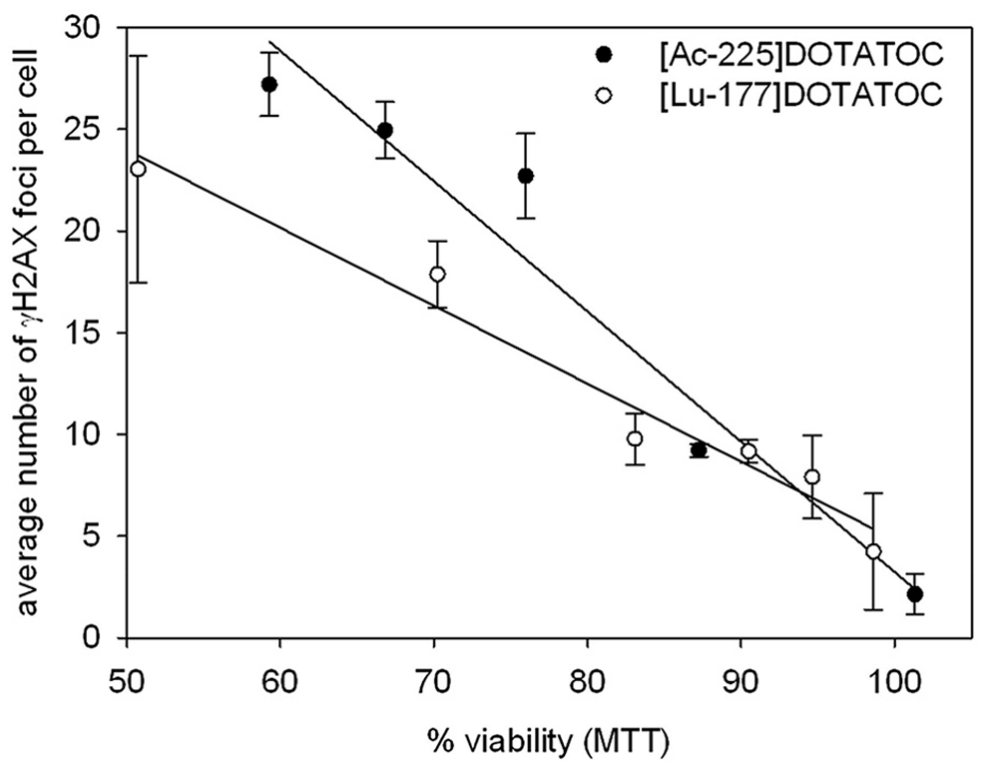
Comparison of cell viability and the number of γH2AX foci in AR42J cells at 48 h after incubation with ^225^Ac-DOTATOC and ^177^Lu-DOTATOC.

DNA double-strand breaks (DSB), localized by γH2AX staining, increased with higher concentrations of ^225^Ac-DOTATOC (2.5 to 10 kBq/ml) and ^177^Lu-DOTATOC (0.6 to 10 MBq/ml) after 24 and 48 h. The spontaneously detected number of γH2AX foci in untreated cells was 2 to 5 on average. The data following incubation with equitoxic doses of ^225^Ac-DOTATOC and ^177^Lu-DOTATOC, extracted from the cell viability data, showed similar levels of DSB; although with the tendency for the less complex γH2AX staining to be detectable after treatment with ^177^Lu-DOTATOC compared to ^225^Ac-DOTATOC. The average number of γH2AX foci was 25 at 10 kBq/ml ^225^Ac-DOTATOC and 22 at 10 MBq/ml ^177^Lu-DOTATOC 48 h after incubation. The maximum number of γH2AX countable was 50. Only in case of ^225^Ac-DOTATOC at doses higher than 10–15 kBq/ml pan-nuclear staining was detectable (Fig. 3). Pan-nuclear staining of γH2AX was reported previously in IMR90 fibroblasts exposed to the ß-particle emitter ^32^P [21]. In addition, nuclear-wide γH2AX staining was observed after local heavy ion-irradiation and occurred in an ATM-dependent manner, correlating with the amount of clustered DNA damage induced [22]. This may also explain the observed pan-nuclear distribution detected in AR42J cells after treatment with high dose ^225^Ac-DOTATOC. Plotting the average number of γH2AX foci per cell over cell viability after ^225^Ac-DOTATOC and ^177^Lu-DOTATOC incubation, respectively, two linear curves were obtained. At the same viability level, a higher number of DSB per cell were found after incubation with ^225^Ac-DOTATOC (Fig.4). This might have an effect on cell death or survival at later time points, due to the powerful induction of apoptosis after severe DNA damage. However, it demonstrates that the DSB from alpha particles are of similar down-stream potency compared to DSB generated from a correspondingly higher dose of beta-particles.

**Figure 5:**
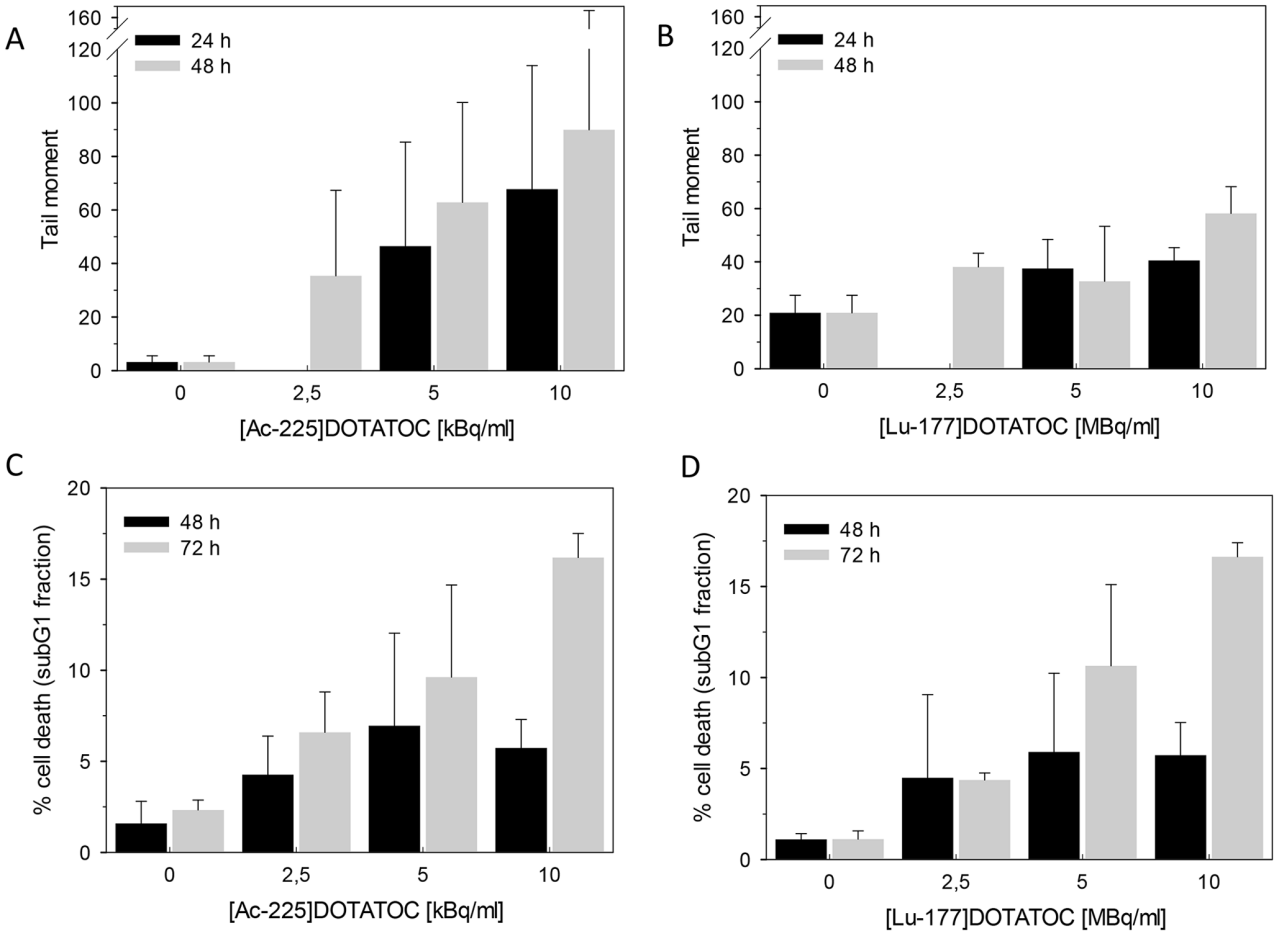
Single-cell gel electrophoresis (alkaline comet assay) of AR42J cells at 24 h and 48 h after incubation with ^225^Ac-DOTATOC (A) and ^177^Lu-DOTATOC (B). DNA damage was calculated by the tail moment, the most frequent used parameter of comet features. SubG_1_ fraction of AR42J cells at 48 and 72 h after incubation with ^225^Ac-DOTATOC (C) and ^177^Lu-DOTATOC (D).

The alkaline comet assay, reflecting DNA single-strand breaks and DSB and alkali-labile sites in the DNA, showed similar results: higher doses of ^225^Ac-DOTATOC resulted in an increased level of DNA damage (Fig. 5). At equitoxic doses calculated from the results of the MTT viability assay, ^177^Lu-DOTATOC showed the tendency for less tail moment values compared to ^225^Ac-DOTATOC. Generally, the variation of the tail moments were higher in the alpha treated samples, possibly reflecting the fact that radiation exposure is less homogenous due to the physical properties of alpha particles. A relative high background tail moment for Lu-177 irradiated cells might be explained by cross irradiation over different wells.

### Cell Cycle Analysis and Induction of Apoptosis

**Figure 6:**
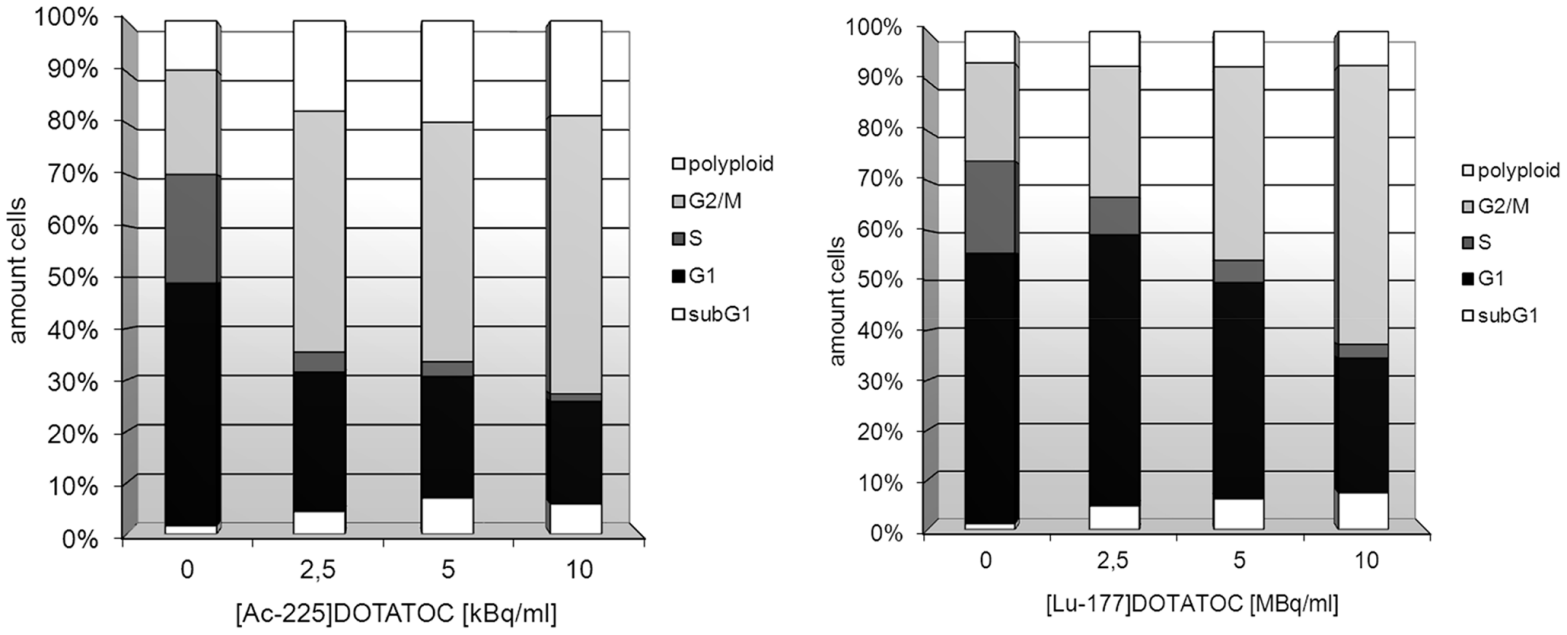
Cell-cycle distribution of AR42J cells at 48 h after incubation with ^225^Ac-DOTATOC (left) and ^177^Lu-DOTATOC (right) (SD <15%).

Cell cycle studies showed an increase in the percentage of tumor cells in G_2_/M phase up to 60% already at 24 h (data not shown) and after 48 h of incubation with 2.5, 5, and 10 kBq/ml ^225^Ac-DOTATOC. Further, an increasing amount of polyploid cells could be detected after 48 h incubation with ^225^Ac-DOTATOC. Even at low concentrations of ^225^Ac-DOTATOC (2.5 MBq/ml), the amount of cells in G_1_ phase was decreased (Fig. 6). At a higher dose the G_2_/M cell cycle blockage was also detectable (decrease of S-Phase fraction). After 48 and 72 h of incubation with both radioconjugates, apoptosis as determined by subG_1_ flow cytometry was detectable (Fig. 5).

### Biodistribution of ^225^Ac-DOTATOC and ^177^Lu-DOTATOC

Biodistribution studies 48 h after injection of tumor bearing nude mice showed nearly the same binding/uptake of ^225^Ac-DOTATOC and ^177^Lu-DOTATOC in the AR42J tumor of 7.5% ID/g. Less uptake of both compounds was found in the kidneys (5.8% ID/g for ^225^Ac-DOTATOC and 1.5% ID/g for ^177^Lu-DOTATOC). In general, higher uptake values were found in all organs for ^225^Ac-DOTATOC compared to ^177^Lu-DOTATOC. Unexpectedly, high liver uptake (>10% ID/g) was observed for ^225^Ac-DOTATOC. For this particular case, increased liver uptake is likely due to approximately 10% free ^225^Ac that was contained in the radiolabeled product, compared to only 0.05% ID/g detectable for ^177^Lu-DOTATOC. Other measured organs, i.e. heart and muscle, showed no or only marginal uptake (<1% ID/g) of ^225^Ac-DOTATOC and ^177^Lu-DOTATOC 48 h after injection of the radionuclides (data not shown).

### Generation of DSB and Growth Kinetics of Neuroendocrine Xenograft Tumors after Treatment with ^225^Ac-DOTATOC and ^177^Lu-DOTATOC

**Figure 7:**
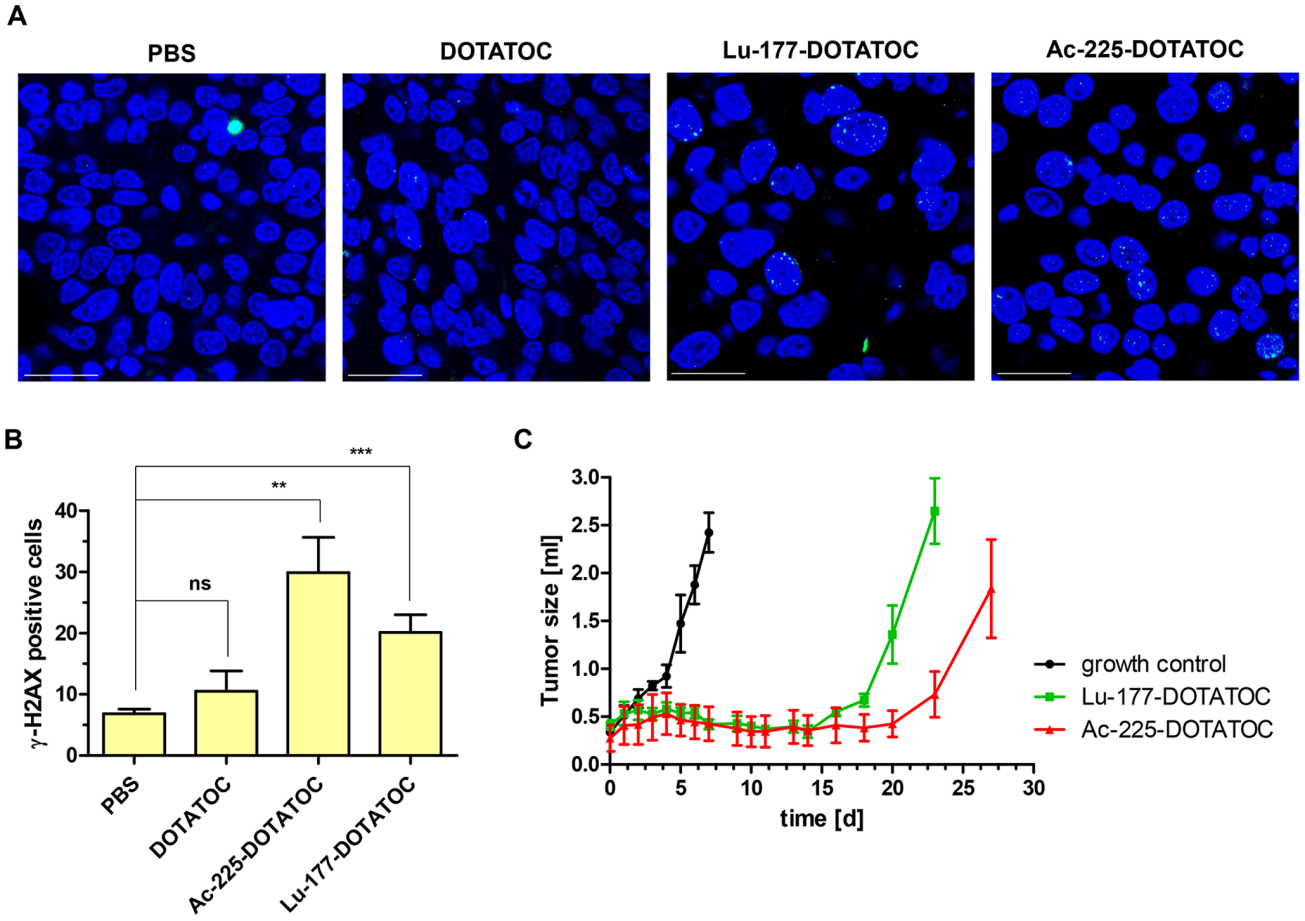
Immunofluorescence staining of DNA double strand breaks (γH2AX) in AR42J tumors after treatment with 47 kBq ^225^Ac-DOTATOC, 30 MBq of ^177^Lu-DOTATOC, 1 µg DOTATOC (unlabelled), or PBS. (**A**) Representative images, (B) and quantification of γH2AX- positive cells. Scale bar (white) corresponds to 25 µm. (C) Growth delay after treatment with equitoxic doses of ^225^Ac-DOTATOC (n = 3, 44kBq/mouse) and ^177^Lu-DOTATOC (n = 4, 34 MBq/mouse) versus untreated control (n = 4).

The number of cells with at least one clear γH2AX focus was significantly elevated in tissue sections 48 h after treatment for both ^225^Ac-DOTATOC and ^177^Lu-DOTATOC compared to nonradioactive controls, whereas overall ^225^Ac-DOTATOC treated tumors showed a higher fraction of cells with γH2AX foci (Fig. 7 A and B). A non-significant increase was also noted for unlabeled DOTATOC compared to the non-treated control (Fig. 7 A and B). Following a single treatment of tumor-bearing mice with equitoxic doses of ^225^Ac-DOTATOC and ^177^Lu-DOTATOC, we observed a strong growth delay of 20 and 15 days, respectively, compared to the corresponding controls (Fig. 7C). Comparison of tumor growth with a linear statistical model showed differences of the three groups within the first 7 days after treatment (p = 0.006). Afterwards, comparing the time curves for the ^225^Ac-DOTATOC and ^177^Lu-DOTATOC groups, this model did not reach statistical significance (p = 0.174). During the observation period no weight loss or clinically evident toxicity was noted. This indicates that the radionuclides at dose levels of approx. 40 kBq ^225^Ac-DOTATOC and approx. 30 MBq ^177^Lu-DOTATOC exert tumor growth inhibiting effects without significant systemic short-term toxicity.

## Discussion

Internal radiotherapy with particle-emitting isotopes coupled to somatostatin analogues such as DOTATOC has become a widely accepted therapeutic option for neuroendocrine tumors. Radiolabelled DOTATOC is rapidly distributed throughout the body and excreted renally. Rapid binding and uptake into tumor tissue and long retention are the main reasons for their high therapeutic effectiveness. These pharmacokinetic properties are ideal for longer lived isotopes where decay of unbound radioactivity mainly occurs after excretion. However, cure is seldom achieved and aspects of dose limiting activities and the potential benefit of other isotopes are not exactly known. We have hypothesized that at least in a subset of patients with radio-resistant tumors the use of alpha emitters might be a further therapeutic option [9]. With its higher LET, alpha-particles are likely to overcome treatment resistance, although the biological response to radiation of neuroendocrine tumors might be generally heterogeneous due to clonal differences in death pathways and DNA damage repair capacity.

Direct detection of DNA damage is one of the most important biological markers in assessment of pharmacodynamic effects of tumor treatments. DSB may be detected on a molecular level by staining of the early DNA damage response marker, phosphorylated histone 2AX (γH2AX). Of note, γH2AX has been shown to be more sensitive as a marker of DSB than the Comet assay, and has been recommended as a surrogate clinical marker of DNA damage [23]. It is also useful to follow up DNA repair since persisting, non-repaired DSB can be visualised by persisting γH2AX foci [24].

By comparing the therapeutic isotopes ^225^Ac and ^177^Lu, effects due to differences in dose rates that typically strongly influence radiobiological responses are minimized. For example, the temporal cellular responses to radiation from these isotopes are more comparable than to comparisons between the short-lived alpha emitter ^213^Bi (half-life: 46 min) and longer-lived beta isotopes [25], [26]. Furthermore, we accounted for the expected significant differences in cytotoxicity in the model system using the SSR positive cell line AR42J. A comparison between these isotopes was therefore conducted on equitoxic dose levels of the alpha- and beta-emitter.

Comparative cytotoxicity assessment demonstrated that a factor of approximately 700 applies between ^177^Lu and ^225^Ac. On this basis and taking into account the different physical properties, a relative biological effectiveness of approximately 5 is calculated (Fig. 2, Table 1), thereby accurately predicting downstream killing effects. In fact, DNA damage as measured by counting γH2AX-foci is similarly related to equitoxic doses of both radioisotopes. Differences between ^225^Ac-DOTATOC and ^177^Lu-DOTATOC as to DSB mediated apoptosis and cell death were relatively low. Nevertheless, a fraction of cells are more heavily damaged at high activities of the alpha emitter, as demonstrated by greater tail moment in the comet assay (Fig. 5), the higher fraction of polyploid cells on cell cycle analysis (Fig. 6) and *in vivo* in a slightly higher tumor control rate (Fig. 7).

**Table 1:** Physical properties of the radio-isotopes and calculation of their relative biological effectiveness on basis of 48 h *in vitro* cytotoxicity.

Comparison of physical properties			
Isotope	alpha particle energy (MeV)	beta particle energy (MeV)	total particle energy/decay
**Ac-225**	5.8		
	6.3		
	7.1		
	8.3	0.66	
			28.16
**Lu-177**		0.147	
			0.147
**Ratio after total decay:**			**192**
**Ratio after 48 decay:**			**131**
**Comparison of cytotoxicity**		ED 50% (kBq/ml)	
Lutetium-177-DOTATOC		10000	
Ac-225-DOTATOC		14	
**Ratio of cytotoxicity:**			**714**
**Relative Biological Effectivness (after 48 h):**			**5**

γH2AX seems to be an important predictor for radiation-induced effects, independent of the radiation quality. For example, a linear relationship between the number of γH2AX-foci and cell viability for both isotopes is evident (Fig. 4). Furthermore, γH2AX phosphorylation in the tumor tissue correlated well with the inhibition of tumor growth (Fig. 7). In line with the *in vitro* experiments unexpected significant differences were not detected between the Ac-225-DOTATOC and the Lu-177-DOTATOC treated groups. However, the efficacy for both treatments over controls was demonstrated. Although reliable subcutaneous growth with small variance is an inherent property of the used animal model and repeated measurement analysis further reduce variance, small differences between both radioactive isotopes cannot be excluded and remain likely. With an *in vivo* tumor doubling time of approximately 2 days, a growth delay of 15 to 20 days equals a tumor control fraction of 90–95% of tumor cells achieved by single treatment with either 44 kBq ^225^Ac-DOTATOC or 34 MBq ^177^Lu-DOTATOC. We can speculate, that differences in DNA repair and apoptosis pathways in some tumor tissues possibly increase the therapeutic effect of alpha emitters over beta emitters (for example in hypoxic/radioresistant cells). *In vivo* dosimetry cannot be calculated reliably since time activity information on the tumors was not obtained. On assumption that half of the number of decays measured at the single time point (48h after treatment) add to the tumor dose, a rough dose estimation could be made from a tumor tissue activity concentration of 45 kBq * 0.075 ID/g = 3.4 kBq/g for ^225^Ac-DOTATOC and 35 000 kBq/g * 0.075 ID/g = 2625 kBq/g for ^117^Lu-DOTATAOC. These activity concentrations are thus multiplied by 0.5 (some of the tumor bound activity will be released and some will decay at time points where an effect on tumor is no longer expected) and energy is calculated from numbers of decays and particle energy/decay [27], [28]. After conversion of the respective units (electronvolts to joules with 1.6E-13 J/MeV, and g to kg,) such an estimate would deliver approximately 10 Gray of alpha particle dose and 25 Gy of beta particle dose. This moderately decreases the *in vivo* RBE of alpha radiation compared to values measured *in vitro*. After a single treatment most of the radiation dose to tumors and organs are delivered within the first few days depending on the biologic half-life of radiolabeled somatostatin analogue that add to the physical half-life (resulting in the effective half-life). Tumor growth after one single treatment was delayed well above two half-lives of the radioisotopes, reflecting long-term effects after initial radiation. Integrity of the peptide and its link to the radioisotope is ensured over several days, but upon decay the daughter nuclides from ^225^Ac are released and distributed with their own pharmacokinetics [29]. Nevertheless, due to rapid initial distribution and diffusion into tissue of the peptide together with high affinity binding to their receptors and high internalization into tumor cells delivery of radiation is determined significantly by the early component of the biodistribution.

Compared to other cell lines from which data are available, AR42J cells do not display great sensitivity towards ^225^Ac. Activity concentrations of up to 2 orders of magnitude higher than in other studied systems are required to devitalize 50% of cells [6], [29]. However, an exact comparison is not possible due to different assays and time points. The AR42J cell line retained the p53 transactivation activity and apoptosis execution and represents, therefore, a reasonable model for our studies [30]. This was further corroborated by our initial experiments showing an IR-dependent induction of the DDR in AR42J cells with activation of p53 and repair of DSBs as reflected by γH2AX kinetics (Fig. 1). Alpha resistance in leukemia cells has been induced at low doses and was attributed to several factors including reduced apoptosis, G2 checkpoint maintenance and increased DNA repair [31]. Cell cycle analysis after ^225^Ac-DOTATOC or ^177^Lu-DOTATOC treatment points to a similar direction with a higher fraction of G2/M cells for alpha irradiation at low doses compared to beta irradiation (Fig. 6).

In contrast to external beam radiation, internal radiotherapy displays several differences that influence its mechanism of action. In addition to generally more inhomogeneous spatial dose distribution for example within tumor tissue, temporal dose distribution is neither discrete nor is it fractionated. It is a continuous radiation with exponential decline according to the half-live of the therapeutic radioisotope. Thus, radiation response including adaptive responses like repair and cell cycle arrest might be different. For optimal application of internal radiotherapy applied activities and its fractionation into different cycles cannot be easily concluded from experience with external radiation. To establish the most beneficial application regimens and possible combination of different isotopes and combination of internal and external radiation γH2AX quantification might play a key role.

Pan nuclear staining might be a general stress response due to radiation that differs from localized response to a limited number of DNA-damage [21]. It will complicate quantification of γH2AX foci at higher concentrations. However, its investigation might add information to the spatial dose distribution of higher doses, which might be important for the more inhomogeneous dose distribution of alpha radiation.

For further development of internal carrier-driven radiotherapy, a profound understanding of the underlying mechanism is essential. Especially, with increasingly successful use of the relatively long-lived beta emitter ^177^Lu, the additional potential of other therapeutic isotopes such as the long-lived generator nuclide ^225^Ac needs to be characterized. Our model system displayed similarities between the two radiation types at therapeutic active doses with a tendency of delayed DNA damage for the alpha emitter. In this model we have shown high tumor control rate *in vivo* after single treatment with both isotopes mediated by somatostatin receptor targeting. Interestingly, molecular effects like apoptosis and *in vivo* effects like tumor growth were accurately correlated to the number of DSB detected by γH2AX. Therefore, we conclude this marker can serve as an early key surrogate marker for therapeutic effects following internal radiotherapy.
